# The Diagnostic Value of Onconeural Antibodies Depends on How They Are Tested

**DOI:** 10.3389/fimmu.2020.01482

**Published:** 2020-07-14

**Authors:** Raquel Ruiz-García, Eugenia Martínez-Hernández, Albert Saiz, Josep Dalmau, Francesc Graus

**Affiliations:** ^1^Immunology Department, Centre Diagnòstic Biomèdic, Hospital Clínic, Barcelona, Spain; ^2^Neuroimmunology Program, Institut d'Investigacions Biomèdiques August Pi i Sunyer (IDIBAPS), Barcelona, Spain; ^3^Neurology Service, Hospital Clinic, University of Barcelona, Barcelona, Spain; ^4^Catalan Institution of Research and Advanced Studies (ICREA), Barcelona, Spain; ^5^Neurology Department, University of Pennsylvania, Philadelphia, PA, United States

**Keywords:** autoantibodies, paraneoplastic neurological syndromes, line blot, onconeural antibodies, diagnostic tests

## Abstract

Detection of onconeural antibodies is important because establishes a definitive diagnosis of paraneoplastic neurological syndrome (PNS). The recommended method for diagnosis of onconeural antibodies is by immunohistochemistry on rodent brain sections and confirmation of results by immunoblot. However, in many diagnostic laboratories samples are only tested with commercial line blots. In this study we inquired whether this change in diagnostic methodology (line blot alone vs. combined immunohistochemistry and line blot) would affect the results. Among 439 samples examined by immunohistochemistry and a commercial line blot (Euroimmun, Lübeck, Germany) 96 (22%) were positive by line blot, and their clinical information was reviewed. Onconeural antibodies were detected by both assays in 46/96 (48%) patients (concordant group) whereas 50 (52%) were only positive by line blot (discordant group). In the concordant group 42/46 (91%) patients had a definite diagnosis of PNS whereas in the discordant group only 4/50 (8%) had PNS (*p* < 0.00001). None of the 14 patients with ZIC4 antibodies and 1/13 (8%) with Yo antibodies demonstrated only by line blot had PNS. These findings show a robust diagnostic value of combined diagnostic techniques, and both should be used to demonstrate onconeural antibodies, If antibody testing is performed only with line blot assay, positive bands should be confirmed by rodent brain immunohistochemistry. For ZIC4 or Yo antibody testing, line blot positivity with negative immunohistochemistry has no diagnostic significance, and for the rest of onconeural antibodies the predictive diagnostic value is low.

## Introduction

Paraneoplastic neurological syndromes (PNS) include a group of neurological disorders in which an immune response against an underlying systemic tumor is misdirected to the nervous system causing the clinical manifestations (1). Most PNS associate with onconeural antibodies against intracellular antigens (2). Since their initial description it was acknowledged that onconeural antibodies could occur in 5–15% of patients without cancer or in cancer patients without PNS (2). Despite these limitations the detection of onconeural antibodies is important because (1) establishes a definitive diagnosis of PNS, (2) assists in the differential diagnosis of atypical clinical syndromes, and (3) directs the search of potentially involved tumors (2).

Initial studies on onconeural antibodies showed high specificity for distinct syndromes and tumors. These studies recommended onconeural antibody testing with rat brain immunohistochemistry and confirmation of results by immunoblot (2). However, when testing for onconeural antibodies became widely available several retrospective studies showed that the diagnostic value of these antibodies was lower than expected, resulting in a substantial number of false positive and negative results that clearly affect patient care (3–7). There are two potential reasons for this problem; one is related to the type of disease (rare) and the size of population investigated (larger than needed). It is known that the lower the prevalence of a disease, the higher frequency of false results, particularly if a test is done indiscriminately without solid clinical reasoning (8). Careful clinical selection of patients that need to be tested for onconeural antibodies is the best approach to circumvent this limitation (5, 7). The second reason is likely related to the type and extent of techniques used to demonstrate the onconeural antibodies. Commercialization of diagnostic tests has favored automated testing of multiple antibodies simultaneously (e.g., line blot) and may perceive additional studies such as brain immunohistochemistry, as redundant, time consuming, and cost increasing. Here we postulated that this limited testing approach greatly contributes to false positive results. For this we assessed the frequency of PNS in patients with onconeural antibodies only demonstrated by line blot and compared it with the frequency of PNS in patients in whom the onconeural antibodies were confirmed by two diagnostic tests, line blot, and brain immunohistochemistry.

## Methods

### Patients and Samples

We reviewed the serum results from 2,437 patients that were routinely screened for the presence of onconeural antibodies between October 2016 and August 2019 by indirect immunohistochemistry on rat cerebellar tissue, and selected 439 (18%) that were also tested by a commercial line blot assay (Euroimmun, Lübeck, Germany). In these 439 patients the line blot was used for the following reasons: (1) confirm immunohistochemical studies suggesting the presence of an onconeural antibody, (2) specific request of the referring physician, or (3) confirm results from other laboratories, particularly when those results did not match with patients' symptoms.

Clinical data were collected from patients with positive line blot assay including the following onconeural antibodies: Hu (ANNA1), Yo (PCA1), CV2 (CRMP5), Ri (ANNA2), Ma2, Tr (DNER), Zic-4, SOX1, and amphyphisin. Patients with positive bands for GAD65, titin, and recoverin antibodies included also in the same kit were excluded from the analysis. Clinical information not available in our files was obtained by phone interview with the referring physicians.

Patients were defined as definite PNS if they had: (1) a neurological syndrome of unexplained cause associated with cancer and presence of an onconeural antibody by line blot, or (2) a classical PNS syndrome (2) (for example sensory neuronopathy or limbic encephalitis) associated with cancer or an onconeural antibody. Patients with isolated neurological symptoms of unclear etiology (for example diplopia, dizziness, or cramps), or with neurological syndromes with an alternative diagnosis (for example alcoholic polyneuropathy) were not considered to have PNS regardless of the presence of cancer or onconeural antibodies.

The study was approved by the ethics committee of the Hospital Clínic. Patient information was anonymized prior to analysis. Written inform consent was not required as the study was observational and the onconeural antibodies were requested as part of the routine diagnostic work-up.

### Rat Cerebellar Immunohystochemistry

Immunohistochemistry was performed as previously described (9). Briefly, adult male Wistar rats were anesthetize and perfused with 0.9% saline solution followed by 2% paraformaldehyde. The cerebellum was further fixed with 2% paraformaldehyde for 4 h and cryoprotected with 20% sucrose in phosphate buffered saline (PBS) overnight. The samples were frozen in methylpropane chilled in liquid nitrogen. Frozen section were air-dried for 30 min and sequentially incubated with 10% normal goat serum for 20 min, patient's serum (diluted 1:500) for 3 h at 37°C, biotinylated goat anti-human IgG for 30 min, and the avidin—biotin immunoperoxidase complex (Vector Labs, Burlingame, CA, USA) for 30 min. The reaction was developed with 0.05% diaminobenzidine (Sigma, St. Louis, MO, USA) with 0.01% hydrogen peroxide in PBS with 0.5% Triton X-100.

### Line Blot

Serum samples were tested by the commercial immunoblot kit EUROLINE Paraneoplastic Neurological Syndromes 12 Ag (DL 1111-1601-7 G; Euroimmun, Lübeck, Germany) following the manufacturers' instructions at serum dilution 1/100. Test strips were scanned and evaluated for band intensity of Hu (ANNA1), Yo (PCA1), CV2 (CRMP5), Ri (ANNA2), Ma2, Tr (DNER), Zic-4, SOX1, and amphiphysin using the EUROLineScan software (Euroimmun Lübeck, Germany). Following the manufacturer's recommendations, onconeural antibody band intensity values ≤10 were considered as negative. Intensity values between 11 and 25 were considered low positive, between 26 and 50 positive, and >50 strong positive.

## Results

Ninety-six out of 439 (22%) patients tested by line blot showed a reactive band suggesting one or more of the indicated onconeural antibodies. In total, 118 bands were identified in the 96 sera, 78/96 (81%) sera had one band, and 18/96 (19%) had two or more bands. In 46 (48%) of 96 patients, the positive line blot result was concordant with the immunohistochemistry findings, and in the other 50 (52%) the result was discordant (negative immunohistochemistry).

**Figure 1:**
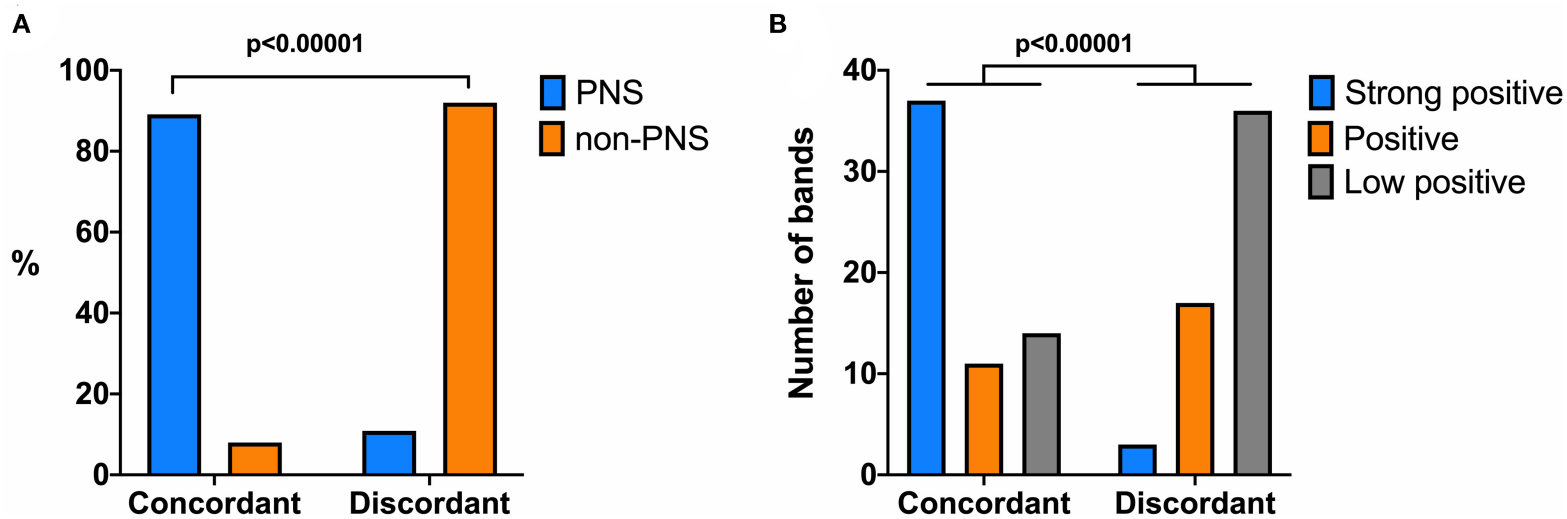
Associations of diagnosis of paraneoplastic neurological syndrome (PNS) **(A)** and band intensity in the line blot **(B)** with samples with onconeural antibodies detected by line blot and immunohistochemistry assays (concordant) or only by line blot (discordant). Chi-square test used to show statistical significant differences.

Patients with concordant results were more likely to have PNS than those with discordant results (42/46 [91%] vs. 4/50 [8%]; *p* < 0.00001; Figure 1A). In the concordant group 42/46 (91%) had a definite diagnosis of PNS and 37 (90%) of them had cancer. The neurological diagnosis of the remaining four patients, two had myasthenia, thymoma, and CV2 (CRMP5) antibodies, and two had brain metastasis in the setting of lung cancer and SOX1 and Ma2 antibodies (Table 1). In these four patients the onconeural antibody reflected the presence of the underlying cancer but they were not related with the neurological syndrome.

**Table 1:** Neurological syndrome and tumor type in patients with onconeural antibodies detected by immunohistochemistry and line blot assays (concordant group) or by line blot with negative immunohistochemistry (discordant group).

	**Concordant *N* = 46**	**Discordant *N* = 50**	***p***
Median age in years (range)	62 (25–84)	58 (19–84)	n.s.
Male/Female	24/22	27/20	n.s.
**Definite paraneoplastic syndrome**	**42**	**4**	<0.00001
Sensory neuropathy	11	0	
Paraneoplastic cerebellar degeneration	8	3	
Limbic encephalitis	8	0	
Paraneoplastic encephalomyelitis	4	0	
Brainstem encephalitis	3	0	
Lambert Eaton myasthenic syndrome	2	1	
Chorea	2	0	
Other^a^	3	0	
**Non-paraneoplastic syndrome**	**4**	**46**	<0.00001
Neuromuscular	0	10	
Epilepsy	0	6	
Immune-mediated	2^b^	6^c^	
Brain metastasis	2	1	
Psychiatric	0	2	
Metabolic encephalopathy	0	2	
Neurotoxicity of ICI	0	3	
Other	0	16	
**Cancer**	**42**	**15**	<0.00001
Small cell lung cancer (SCLC)	20	3	
Non-SCLC	8	3	
Breast	4	0	
Thymoma	3	2	
Ovary	2	2	
Testis	2	0	
Hodgkin lymphoma	1	0	
Other	2	5	

*ICI, immune checkpoint inhibitors; n.s., not significant*.

a *Intestinal pseudo-obstruction; stiff-person syndrome; epilepsia partialis continua*.

b *Myasthenia and thymoma*.

c *Polyneuropathy associated with MAG antibodies and Sjögren syndrome, gluten ataxia, myasthenia, stiff-person syndrome, Morvan syndrome*.

In the discordant group, only 4/50 (8%) patients had a definite diagnosis of PNS. All had lung cancer (small cell lung cancer [SCLC] in three), three with paraneoplastic cerebellar degeneration (PCD) and SOX1, CV2 (CRMP5) and Yo (PCA1) antibodies, and 1 with Lambert Eaton myasthenic syndrome and SOX1 antibodies. The neurological diagnoses of the other 46 patients of the discordant group are summarized in Table 1. Most, 16/46 (35%) had isolated symptoms (cramps, diplopia) or disorders unrelated with cancer (subarachnoid hemorrhage, spinal arteriovenous fistula).

Patients with concordant results had a higher frequency of cancer than those discordant (42/46 [91%] vs. 15/50 [30%], *p* < 0.00001; Table 1). In the discordant group, 11/15 (73%) patients with cancer had no PNS, and in nine of them the antibody suggested by the line blot assay did not match with the tumor type (Table 2).

**Table 2:** Neurological syndrome, tumor and antibody type in 11 patients with cancer, onconeural antibodies detected only by line blot, and no PNS.

**Patient**	**Neurological syndrome**	**Tumor**	**Antibody**	**Expected result according to cancer type?**
1	Alcoholic neuropathy	NSCLC	CV2 (CRMP5)	Yes
2	Brain metastasis	NSCLC	Yo (PCA1)	No
3	Neurotoxicity ICI	NSCLC	Yo (PCA1)	No
4	Subarachnoid hemorrhage	Ovary	Zic4	No
5	Decreased visual acuity	Ovary	Zic4	No
6	Myasthenia	Thymoma	CV2 (CRMP5)	Yes
7	Morvan syndrome	Thymoma	Yo (PCA1)	No
8	Seizures	Rectum	Yo (PCA1)	No
9	Neuroxicity ICI	Prostate	SOX1	No
10	Neuroxicity ICI	Melanoma	CV2 (CRMP5)	No
11	Decreased visual acuity	CUO	Zic4	No

*CUO, carcinoma of unknown origin; ICI, immune checkpoint inhibitors*.

The intensity of the 118 positive bands identified in the line blot assay was analyzed and compared with the results of the immunohistochemistry and the PNS association. Forty (34%) of 118 bands had strong intensity and 37 (93%) of the corresponding samples showed positive immunohistochemistry. In contrast, 28 (24%) bands were defined as positive, and 50 (42%) as low positive, and the corresponding samples showed positive immunohistochemistry in 11/28 (39%) and 14/50 (28%), respectively (*p* < 0.00001; Figure 1B). Concerning the association with PNS, strong intensity bands occurred in samples of 33/45 (73%) patients with PNS and in 5/51 (10%) patients without PNS (*p* < 0.00001).

We then analyzed the specificity of the individual onconeural antibodies found in the line blot assay for the diagnosis of PNS (Table 3). For 18 (19%) of the 96 samples with more than one band, only the more intense band was considered in the analysis. None of the 14 samples with isolated or predominant ZIC4 bands (band intensity: low positive nine, positive five, strong positive zero) was confirmed by immunohistochemistry, and none of the patients had PNS. Similarly, among 13 patients with discordant anti-Yo results (all line blot positive, immunohistochemistry negative) 12 (92%) did not have PNS (band intensity: low positive seven, positive six, strong positive zero). In contrast, all four patients with concordant Yo-antibody results had PNS (PCD female with breast or ovarian cancer) and the band intensity was strong positive in three and positive in one. For the other antibodies, the frequency of PNS was 50% (9/18) for CV2 (CRMP5), 53% (8/15) for SOX1, 87% (13/15) for Hu (ANNA1), and 59% (10/17) for the remaining [Tr, Ri (ANNA2), and amphiphysin] antibodies detected by line blot assay regardless of the immunohistochemistry result (Table 3).

**Table 3:** PNS diagnosis frequency according to antibody type and concordance between line blot and immunohistochemistry assays.

**Antibody (*N*)**	**PNS**		**Non-PNS**	
	**Concordant *N* (%)**	**Discordant *N* (%)**	**Concordant *N* (%)**	**Discordant *N* (%)**
Zic 4 (14)	0 (0)	0 (0)	0 (0)	14 (100)
Sox1 (15)	6 (40)	2 (13)	1 (7)^a^	6 (40)
Hu (15)	13 (86)	1 (7)^b^	0 (0)	1 (7)
CV2 (18)	8 (44)	1 (6)	2 (11)^c^	7 (39)^d^
Yo (17)	4 (23)	1 (6)^e^	0 (0)	12 (71)
Other (17)^*^	10 (59)	0 (0)	2 (12)	5 (29)

* *Ma2, Tr, Ri, amphiphysin results were pooled due to the low number of positive cases (<10). PNS: paraneoplastic neurological syndrome. Concordant: line blot and immunohistochemistry assays show the same result*.

a *Sample from a patient with lung cancer and brain metastasis*.

b *The sample also had CV2 antibodies concordant with the immunohistochemistry*.

c *The two samples were from patients with thymoma and myasthenia*.

d *All samples had a low positive intensity in the line blot*.

e *Atypical for Yo antibodies: PCD male with lung cancer*.

## Discussion

Our study shows that the type of approach to onconeural antibody testing using line blot, immunohistochemistry, or both techniques (dual approach) strongly influences the accuracy in diagnosing PNS. Indeed, 91% of patients with onconeural antibodies demonstrated by line blot and rat immunohistochemistry had PNS compared to 8% of those with antibodies detected only by line blot. These findings are in line with a recent report showing that only 33% of onconeuronal positive cases by commercial line blots had antibodies detectable with additional techniques (rodent brain immunostaining, or in-house immunoblot) (10). Moreover, 84% of patients only positive by line blot had alternative neurologic diagnoses (e.g., the final diagnosis was not PNS), whereas most patients with dual testing positivity had PNS (10).

In our study, the two antibodies that caused more misdiagnoses were ZIC4 and Yo. Indeed, none of the 14 samples with isolated or predominant positive ZIC4 bands in the line blot was confirmed by immunohistochemistry and none of the patients had PNS. ZIC4 antibodies were initially described in patients with SCLC without PNS and later in a patient with subacute cerebellar ataxia without cancer (11, 12). A subsequent study using an in-house immunoblot with recombinant ZIC4 protein showed that 49 patients with ZIC4 antibodies had PNS related to lung cancer (40 had additional Hu or CV2 antibodies); 11 additional patients with ZIC4 antibodies had SCLC without PNS, but none of 175 patients with non-cancer related neurological disorders or healthy participants had these antibodies (13). Anti-ZIC4 positive samples were not examined by immunohistochemistry to know the concordance between assays. Although the study suggested a tight association between ZIC4 antibodies and PNS or SCLC, the current study using the commercial version of the assay did not reproduce that association; the reasons for this disparity of results are unclear.

As far as Yo-antibody testing is concerned, a potential reason for false positive results is that the line blot uses the paraneoplastic cerebellar degeneration related antigen (CDR) 2 to probe for Yo (PCA1) antibodies (14). CDR2 was once considered the main target antigen of Yo antibodies, but recent studies showed that the main antigen is CDR2L, which has 45% sequence homology with CDR2 (15, 16). In rat brain immunohistochemistry, Yo (PCA1) antibodies react with CDR2L but not with CDR2 (15). Line blots can detect Yo (PCA1) antibodies because CDR2 and CDR2L share epitopes but additionally detects other antibodies (CDR2-restricted) that do not have PNS-specificity; hence the high frequency of false positive results.

The line blot assay fared better for other antibodies; 44% of patients whose samples were positive for CV2, and 87% of those positive for Hu had PNS. Samples with intense reactive bands in line blot were more likely to be from patients with PNS and also to associate with positive immunohistochemistry, compared with samples with weaker reactive bands. The reason for this finding is that line blots are more sensitive to detect low levels of onconeural antibodies than immunohistochemistry, but there are also less specific for PNS. Indeed, low onconeural antibody levels may occur in patients with cancer without PNS, or in a small proportion of healthy controls (17, 18).

A limitation of our study is that we did not systematically test all samples received by line blot and a selection bias could have been introduced in the analysis. The reason for this, is that for many years we have found a good association between onconeural antibodies detected by immunohistochemistry (and confirmed by immunoblot) with PNS. Based on this experience, immunohistochemistry has a leading role in our onconeural antibody testing. However, according to the present results the testing of all samples by line blot would have increase the number of patients with positive onconeural antibodies only detected in the line blot assay and decreased even further the association with PNS. Another limitation is that the low frequency of some antibodies such as Tr (DNER), Ri (ANNA2), and amphiphysin, has prevented to assess the diagnostic value of line blots for each individual specificity.

These limitations, however, do not affect the main findings and implications of our study: Patients suspected to have a PNS related to classical onconeural antibodies should have dual testing with immunohistochemistry and line blot assays. If antibody testing is performed only with line blot assay, positive results must be confirmed by immunohistochemistry. ZIC4 or Yo antibodies only detected with line blot, most likely represent false positive results. As for the rest of antibodies, strong positive intensity bands associate with a positive immunohistochemistry and PNS diagnosis. Positive and low positive intensity bands not confirmed by immunohistochemistry show a low predictive value for PNS diagnosis. Overall, the implications are important because PNS usually preceded the diagnosis of cancer and often have a poor prognosis. Thus, false positive results may lead to unnecessary tests (cancer screening), increased anxiety of patients and families, and inappropriate treatment and prognostic decisions.

## Data Availability Statement

The raw data supporting the conclusions of this article will be made available by the authors, without undue reservation.

## Ethics Statement

The studies involving human participants were reviewed and approved by Ethics committee of the Hospital Clínic. Written informed consent for participation was not required for this study in accordance with the national legislation and the institutional requirements. The animal study was reviewed and approved by European and local regulations (2010/63/UE and RD53/2013).

## Author Contributions

FG designed the study and wrote the manuscript in collaboration with JD, RR-G, EM-H, and AS. RR-G, EM-H, and FG performed laboratory work and/or data analysis. All authors discussed the results and commented on the manuscript.

## Conflict of Interest

JD receives royalties from Athena Diagnostics for the use of Ma2 as an autoantibody test and from Euroimmun for the use of NMDA, GABAB receptor, GABAA receptor, DPPX, and IgLON5 as autoantibody tests. FG receives royalties from Euroimmun for the use of IgLON5 as an autoantibody test and honoraria for Assistant Editor of MedLink Neurology. The remaining authors declare that the research was conducted in the absence of any commercial or financial relationships that could be construed as a potential conflict of interest.
